# Longer Residence of Ecuadorian and Colombian Migrant Workers in Spain Associated with New Episodes of Common Mental Disorders

**DOI:** 10.3390/ijerph16112027

**Published:** 2019-06-06

**Authors:** Elena Ronda-Pérez, José Miguel Martínez, Alison Reid, Andrés A. Agudelo-Suárez

**Affiliations:** 1 Public Health Research Group, University of Alicante, 03690 San Vicente del Raspeig, Alicante, Spain; elena.ronda@ua.es (E.R.-P.); jmartinezma@mc-mutual.com (J.M.M.); 2 Immigration and Health Program, CIBERESP, 28029 Madrid, Spain; 3 Research and Analysis Service IT/EP, MC Mutual, 08037 Barcelona, Spain; 4 Department of Statistics, Technical University of Catalonia, 08028 Barcelona, Spain; 5 School of Public Health, Curtin University, 6102 Bentley, Perth, Australia; 6 Faculty of Dentistry University of Antioquia, 050010, Medellín, Colombia; oleduga@gmail.com

**Keywords:** migration, mental health, work, Spain

## Abstract

The healthy migrant effect and its impact on mental health has been reported in the general population of many countries. Information is limited about its impact on working populations. The aim of this study is to estimate the incidence of common mental disorders over a one-year follow-up period among a cohort of Colombian and Ecuadorian employees in Spain, taking into account the duration of residence and comparing with Spanish-born workers. Data was from the Longitudinal Studies on Immigrant Families Project (PELFI), a follow-up survey of immigrants and Spanish-born workers interviewed in 2015 and 2016. Mental health was assessed using the 12-item general health questionnaire (GHQ-12). Crude and adjusted odds ratios (ORas) for common mental disorders by sociodemographic and employment characteristics were created. There were differences for immigrants with time of residence less than or equal to 15 years (time of residence 11–15 years: ORa = 0.06, 95% CI = (0.26–0.01); time of residence 1–10 years: ORa = 0.06, 95% CI = (0.36–0.01)). There was evidence of a healthy immigrant worker effect, as newer arrivals from Ecuador and Columbia to Spain had a lower incidence of common mental disorders than either the Spanish-born or immigrant workers who had lived in Spain for more than 15 years.

## 1. Introduction

In the last decade of the 20th century, Spain became a primary destination country for migrants in Europe, the majority of whom came from low-income countries, attracted by the expanding economy. In 1998, there were 5.7 million foreign people (12.19% of the population), 10 times more than in 1988. However, by 2008, Spain’s period of growth ended, as a result of the global financial crisis [1]. The labor market was severely affected and suffered job losses, especially in construction and services—the sectors most vulnerable to the recession, and those that employed, with some differences by gender, most of the immigrant population. Most of these immigrants returned to their countries of origin. Between 2009 and 2015, the number of people of any nationality leaving Spain exceeded those who arrived. That trend was reversed in 2015. The current recovery and growth of the Spanish economy has encouraged immigrant arrivals to recover to pre-crisis volumes, and 1,176,836 workers have a work contract (16.2% of all the contracts) [2]. However, the post-crisis situation has resulted in a highly precariat labor market, affecting the entire salaried population [3].

The quality of work (both in term of working conditions and employment arrangements) is an important determinant of mental health [4]. Adverse working conditions that are associated with poorer mental health include job strain (high demand and low control), low social support (low social interaction in the workplace with either colleagues or supervisors) [5], high job insecurity (high probability of losing your job in the near future) [6], and high psychological demands (e.g. job strain, or high demand/low control) [7].

Furthermore, working in poor quality jobs (e.g. long working hours, irregular shifts, working in bending and awkward positions, etc.) has been shown to be more detrimental to mental health than being unemployed [8]. Work for migrant workers is particularly important, as the chance to improve their economic conditions is the main driver for migration [9]. Research conducted in Spain highlights the importance of good working conditions, wherein the mental health of immigrants working in jobs with good employment conditions improved even during the global financial crisis [1].

Other research has shown that the health of migrants declines to that of the host population with increasing duration of residence [10]. Various theories have been suggested as to why this may occur [11]. The first one is acculturation, where with longer residence in a country, the migrant adopts the habits of the host population, including poor food habits, reduced physical activity, etc. A second theory proposes that lack of access to health care services is responsible for the decline in immigrant health. A third theory proposes that poor socioeconomic circumstances associated with the early periods of migration explain the decline in health. This is supported by the findings from the Longitudinal Studies on Immigrant Families Project (PELFI) study, which show that newer migrants to Spain experienced more adverse working conditions that those who had a longer duration of residence [12].

Several studies have compared mental health outcomes between migrant and non-migrant populations in Europe in the aftermath of the global financial crisis. Results tend to be mixed. Pooling the results of all country data from the 2014–2015 European Social Survey showed no difference in well-being between immigrant and native-born populations [13]. Other work found that immigrants in seven European countries had significantly higher levels of depressive symptoms than the native-born population, while in the United Kingdom and Greece they had significantly lower levels [14]. This study did not examine duration of residence, and both studies examined the whole working age population.

Therefore, the objective of this study is to estimate the incidence of common mental disorders over a one-year follow-up period among a cohort of Colombian and Ecuadorian adults employed in Spain, taking into account the duration of residence and comparing the results to those of Spanish-born workers.

## 2. Materials and Methods

### 2.1. Participants

Data are from the PELFI study (Longitudinal Studies on Immigrant Families Project) [15], with a non-probabilistic sample of 250 families (473 adults) surveyed in Alicante and Barcelona. After an initial survey in 2015, two follow-up waves of data were collected from participants in 2016 and 2017. The definition of family was that of the Spanish National Health Survey: persons who occupy a household—or a part of one—with shared consumption and budget (they share food or other items purchased with a common budget), and who have resided together for at least six months at the time of recruitment. Immigrant families were considered to be those in which both parents (father or mother for single-parent families) were born in Colombia or Ecuador. The selection criteria for a family included having at least one son/daughter aged between 12–17 years old, and at least one adult aged 18–65 years who had worked for at least one year in Spain, not necessarily continuously. As a result, for this study we included only the 473 adults aged ≥18 years. Families had to expect to reside permanently in Spain for at least 18 months after being selected for the study. A sample of 50 native-born Spanish families were also included in the study. All families (immigrants and native-born) were recruited from neighborhoods with high levels of foreigners and low levels of economic resources, compared with the total for the respective municipalities.

Sample size was estimated with a Poisson approximation. Three hundred and fifty-nine immigrants and 107 natives were required to detect a minimum relative risk of 1.5 (based on earlier work), assuming an alpha of 0.05 and beta of 0.2, and an attrition rate of 15%. Assuming four family members per family (an estimated two children per family, based on the number of children per woman of foreign origin in Spain, which is 1.54 [2]) and rounding upwards gives 180 immigrant and 50 Spanish families in each city.

Within each family, all of the adults were interviewed in their homes, or at associations or public places in the neighbourhood, by professional, trained interviewers. Data were collected using ad hoc questionnaires (available at https://web.ua.es/es/gi-saludpublica/trabajo-inmigracion-y-salud-en-una-cohorte-de-poblacion-inmigrante-en-espana.html).

The study was approved under the Research Ethics Committee at the University of Alicante (UA-2014-06-26). Confidentiality was guaranteed throughout the process, and the respondents gave their consent to participate in the study, in accordance with the provisions of Spanish Data Protection. All data were rendered anonymously.

### 2.2. Measures

Common mental disorders were measured at the baseline, 2015, and one year later in 2016, using the Spanish-language validated version of the 12-item general health questionnaire (GHQ-12) with four response options [16]. The GHQ-12 is a commonly used instrument in occupational health research, and is a well-validated measure of non-psychotic disorders and patterns of adjustment associated with distress [17]. For each item, a score of 0 (for responses 1 and 2, less symptomatic) or 1 (for answers 3 and 4, more symptomatic) was assigned, and the 12 resulting scores were added together. A score below three indicated good mental health or an absence of common mental disorders, and a score of three or above indicated poor mental health or the presence of common mental disorders [17].

Other variables included in the study were socioeconomic characteristics and employment conditions: sex, age at the first interview (18–40, 34–40, 41–47, and 48–65 years old), occupational social class (manual and non-manual) [18], educational level (no formal education, primary, secondary, or university education), working hours per week (<40 and >40 hours), informal employment (Spanish Social Security system registration: yes or no), shiftwork (yes versus no), exposed to physical demands at the work place (yes versus no), and income level that precludes covering unforeseen expenses (sometimes/always able to cover unforeseen expenses versus never able to cover unforeseen expenses). For migrant workers, we included the time of residence in Spain, categorized as 1 to 10 years, 11 to 15 years, and more than 15 years. To establish categories 1–10, 11–15, and >15, the following strategy was used. First, the sample was split into quartiles, obtaining groups 1–10 (*n* = 19), 11–13 (*n* = 22), 14–15 (*n* = 48), and >15 (*n* = 13). Secondly, the incidence of common mental disorders was obtained in each of the groups. The incidence was 15.8%, 22.7%, 25.0%, and 46.2%, respectively. Due to the similarity in the incidence of groups 11–13 and 14–15, and the small sample size, we decided to create a single group of 11–15 years. Therefore, the final number of categories were 1–10 (*n* = 19), 11–15 (*n* = 70), and >15 years (*n* = 19).

### 2.3. Analysis

We restricted our sample to participants who were employed at the baseline (2015; *n* = 324 (111 Spanish and 213 immigrant)) and reported good mental health (*n* = 130). After comparing results between immigrant participants and those born in Spain, we evaluated the relationship between length of residence in Spain and incidence of common mental disorders. Odds ratios were crude and adjusted by the city of study, sex, age, educational attainment, occupational class, working hours per week, informal employment, standard hours, physical demands, and income level. Logistic regression models were carried out with family-specific random effects. Data were managed and analysed using the statistical software programs SPSS version 15 (IBM, Armonk, NY, USA) and Stata version 10 (College Station, TX, USA).

## 3. Results

The sample consisted of 28 born-in-Spain and 102 immigrant workers (Table 1). Statistically significant differences were observed in age, where the immigrant group was younger (43.1% versus 25.0% of workers aged between 18–40 years, respectively), with a higher percentage of university studies in those born in Spain (42.9% versus 17.6%, respectively) and a higher percentage in the occupational social class (89.1% manual workers in the immigrant population versus 46.4% for those born in Spain). For employment conditions, statistically significant differences were observed in the type of working day, with a higher percentage of irregular working hours for immigrants (39.6% versus 14.3%, respectively); with regard to whether the salary could handle unforeseen expenses, immigrants have a higher percentage of not handling unforeseen expenses (37.6% versus 3.7%, respectively).

Incidence of common mental disorders (Table 2) is more than two times higher in workers born in Spain than immigrants (60.7% compared to 25.0%, respectively; *p* < 0.001). This pattern is consistent for all groups of sociodemographic variables and employment conditions. These differences are approximately five times higher in young people (100.0% versus 25.0%, respectively; *p* < 0.001) and in non-manual workers (53.3% versus 9.1%, respectively; *p* = 0.036). Moreover, poor mental health was higher in Spanish-born workers who did not work in the informal sector, and whose salary was enough to cover unforeseen expenses, compared with the immigrant workers.

These differences maintained the same direction (Table 3), after adjusting for sociodemographic characteristics and employment conditions (adjusted odds ratio (Ora) = 0.07, CI 95% = 0.29–0.02, 1/ORa = 13.65). Immigrant workers were almost 14 times less likely to have common mental disorders compared with Spanish-born workers.

**Figure 1:**
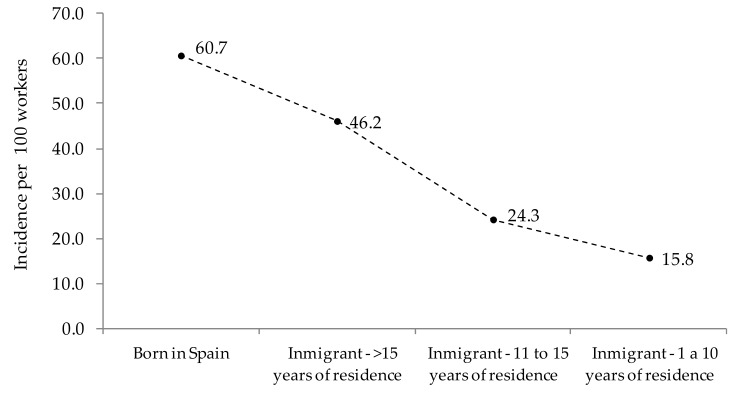
Incidence of common mental disorders by migration status and time of residence in Spain.

Stratifying the immigrant group by time of residence in Spain, a gradient was observed where the incidence of common mental disorders decreased with less residence time in Spain (Figure 1). That is, the incidence of common mental disorders of immigrants with longer duration of residence in Spain was similar to that of the Spanish-born population.

Differences in the incidence of common mental disorders between Spanish-born and immigrant workers were maintained after adjusting by sociodemographic characteristics and employment conditions (Table 3). The results showed no statistically significant differences between workers born in Spain and immigrants with a residence time longer than 15 years (ORa = 0.23, 95% CI = 1.50–0.03, 1/ORa = 4.41). However, there were differences for immigrants with time of residence less than or equal to 15 years: time of residence 11–15 years, ORa = 0.06, 95% CI = 0.26–0.01, 1/ORa = 17.25; time of Residence 1–10 years, ORa = 0.06, 95% CI = 0.36–0.01, 1/ORa = 17.25. Therefore, immigrants who had lived in Spain for one to 10 years or 11–15 years had an almost 18-fold decreased risk of common mental disorders compared with Spanish-born workers (Table 4).

## 4. Discussion

We examined the change to common mental disorders among immigrant and Spanish-born workers with good mental health at the baseline study. Immigrant workers were more likely to work in jobs with worse employment conditions than native-born workers. An exception was long working hours. The incidence of common mental disorders varied by migration status. Immigrant workers who had lived in Spain for less than 15 years had a statistically significantly lower risk of developing common mental disorders than Spanish-born workers did. The incidence of common mental disorders was highest among the Spanish-born workers of all the groups examined. This remained after adjusting for a range of sociodemographic and job quality indicators.

This study found that immigrants who had lived in Spain between 1 and 10 years or between 11 and 15 years had fewer episodes of common mental disorders compared to the native-born population and immigrants who had lived in Spain for more than 15 years. Other studies that have examined the healthy migrant effect often report a decline in the migrants´ health between 5 and 10 years post-arrival in the host country [19]. Immigrants from Columbia and Ecuador tend to migrate to Spain to improve their social and economic conditions [20]. They speak the same language as that spoken in the host country and by the majority of their workmates, which facilitates their access to social support at work, and more generally their integration into Spanish society; these are factors that have been found to be important to the health of working migrants in Sweden. [21]. Other factors improve the settlement experience of Latin American migrants in Spain. They are entitled to Spanish nationality after only two years of residency, and do not have to renounce their original nationality in order to apply for Spanish nationality [20]. For all immigrants in this current study, the incidence of new episodes of common mental disorder was 25.5%. This is similar to that reported for immigrants in 2011 (34.1, 95% CI: 27.8–40.1), which was measured during the peak of the economic crisis [1]. This latter study included all immigrants to Spain, including those who had lived there for longer than 15 years.

New episodes of common mental disorder were highest among the Spanish-born workers in this current study. The two waves of PELFI were undertaken in 2015 and 2016, at the end of the global financial crisis. After 2009, the Spanish labour market saw job losses in construction and services, and an increase in the unemployment rate to 26% in the fourth quarter of 2012 [1] . Other research has reported a prevalence of 17.0% to 22.7% of common mental disorders, measured using the GHO-12, among Spanish men and women, respectively [22]. An increase in the prevalence of common mental disorders was reported in men (prevalence ratio (PR) 1.15, 95% confidence interval (95% CI): 1.04–1.26), but not women (PR 0.92, 95% CI: 0.87–0.98). The increase was greatest in men aged 35–54 years, with primary and secondary education, and from semi-qualified social classes [22]. This study included all Spanish men and women aged 16–64 years, unlike the current survey that included only those who were currently working, which may explain the higher incidence of common mental disorders observed in this current study.

Relationships between declining mental health and duration of residence in the host country have been found to vary among migrant populations. Work from Canada examining immigrants who arrived between 1991 and 2000 found poorer self-reported health among those who had lived in Canada for more than 10 years. Furthermore, this study found statistically significant differences in self-perceived worker stress and total number of hours worked between the two groups of immigrants [11]. A linear trend in poor general health, depression, and increasing duration of residence was found among the mothers of infants in the United Kingdom. No independent association was reported for alcohol consumption, antenatal care, or length of residence [23]. These findings question the acculturation theory, which suggests that adverse lifestyle factors are taken up by the migrant with increasing duration of residence, thus explaining their decline in health. This idea of acculturation towards a mainstream population has been suggested by others as being subtly and dangerously ethnocentric [24].

The healthy migrant effect in some countries, e.g. Canada and Australia, may be an artefact of the selection process of migration. In those countries, a skilled migration scheme exists, and migrants are selected to migrate based on their skills, qualification, and language abilities, rather than being self-selected. In addition, all migrants must undergo a physical health examination [25]. This is not the case for Spain, where there is self-selection of migrants. Despite this, we found a healthy migrant effect among migrants to Spain in this current study.

Our research has some limitations that must be taken into account when interpreting the results. First, we only included migrants from Colombia and Ecuador. Both countries are among the leading non-European Union countries from which workers immigrate to Spain, and they share similar emigration patterns [2]. Therefore, they cannot be generalized to migrants overall. A recent report about the integration of immigrants in Spain in the period 2007–2015 showed the advantage of Latin American persons compared with other nationalities in terms of language, family reunification policies, and naturalization facilities, among others [26]. In addition, we have to consider that participants are economic migrants, and our results will not be the same as other groups, for example refugees, whose mental health issues could be different [27]. The second limitation is related to the small sample size. Even though we found significant differences for most associations, the limited number of workers included in the sample did not permit us to do more specific statistical analysis—for example, stratifying by gender [28]. However, our results showing a higher incidence of common mental disorder among the Spanish-born, although based on only 14 participants, concurs with recent work from Australia, wherein Australian-born workers working in insecure jobs had poorer mental health compared with immigrant workers [29].

The study’s strengths include the opportunity to analyze information about a hard-to-reach population, with a one-year follow up and no losses (all the workers with good mental health at the baseline participated in the second survey). There remain issues around the validity of comparing self-reported measures between different migrant and ethnic groups [30], but this has been overcome using the GHQ-12 instrument, which has been validated in the Spanish spoken in Colombia [31].

## 5. Conclusions

In this study, we compared the incidence of common mental disorders among immigrants from Ecuador and Colombia with the Spanish-born population. There was evidence of a healthy immigrant worker effect, as newer arrivals from Ecuador and Columbia had a lower incidence of common mental disorders than either the Spanish-born or immigrant workers who had lived in Spain for more than 15 years. Bearing in mind that these findings come from a study with a small number of participants, policies aimed at preserving the mental health of new arrivals and improving the mental health of those born in Spain and immigrants with a longer duration of residence are warranted. Although immigrants from Latin America make up a large proportion of the Spanish workforce, other nationalities also immigrate to Spain. Future research on the mental health of workers should include these other groups to get a truer picture of the mental health of the Spanish workforce.

## Figures and Tables

**Table 1:** Distribution of workers included in the study by socio-demographic characteristics, and employment conditions by migration status.

	Born in Spain		Immigrant		
Variables	*n*	(%)	*n*	(%)	*p*
Socio-Demographic					
Sex					
Women	14	(50.0)	61	(59.82)	0.392
Men	14	(50.0)	41	(40.28)	
Age (years)					
18–40	7	(25.0)	44	(43.1)	0.063
41–47	9	(32.1)	36	(35.3)	
48–65	12	(42.9)	22	(21.6)	
Level of Education					
University studies	12	(42.9)	18	(17.6)	0.028
Secondary studies	13	(46.4)	67	(65.7)	
Primary or no education	3	(10.7)	17	(16.7)	
Occupational social class					
Non-manual	15	(53.6)	11	(10.9)	<0.001
Manual	13	(46.4)	90	(89.1)	
Employment conditions					
Works hours per week					
≤40	19	(67.9)	83	(83.0)	0.109
>40	9	(32.1)	17	(17.0)	
Informal employment					
No	25	(89.3)	78	(78.0)	0.190
Yes	3	(10.7)	24	(24.0)	
Shiftwork					
Yes	4	(14.3)	40	(39.6)	0.013
No	24	(85.7)	61	(60.4)	
Physical demand					
No	18	(64.3)	51	(53.7)	0.389
Yes	10	(35.7)	44	(46.3)	
Salary unforeseen expenses					
No	1	(3.7)	35	(37.6)	<0.001
Yes	26	(96.3)	58	(62.4)	
Total	28	(100.0)	102	(100.0)	

**Table 2:** Number and incidence of common mental disorders by socio-demographic characteristics, employment conditions, and migratory status.

	Born in Spain		Immigrant		
Variables	Number of Cases	Incidence	Number of Cases	Incidence	*p*
Socio-Demographic					
Sex					
Women	7	50.0	12	29.3	0.200
Men	10	71.4	14	23.0	0.001
Age (years)					
18–40	7	100.0	9	20.5	<0.001
41–47	5	55.6	13	36.1	0.449
48–65	5	41.7	4	18.2	0.224
Level of Education					
University studies	8	66.7	5	27.8	0.061
Secondary studies	6	46.2	15	22.4	0.092
Primary or not studies	3	100.0	6	35.3	0.074
Occupational social class					
Non-manual	8	53.3	1	9.1	0.036
Manual	9	69.2	24	26.7	0.004
Employment conditions					
Works hours per week					
≤40	11	57.9	21	25.3	0.012
>40	6	66.7	4	23.5	0.046
Informal employment					
No	15	60.0	16	20.5	<0.001
Yes	2	60.7	10	41.6	0.569
Shiftwork					
No	14	58.3	14	23.0	0.040
Yes	3	75.0	11	27.5	0.088
Physical demand					
No	11	61.1	10	19.6	0.002
Yes	6	60.0	13	29.5	0.139
Salary unforeseen expenses					
No	0	0.0	11	31.4	1.000
Yes	16	61.5	10	17.2	<0.001
Total	17	60.7	26	25.5	0.001

**Table 3:** Association (crude odds ratio (ORc) and adjusted odds ratio (ORa) with 95% confidence intervals (CI)) of common mental disorders of immigrants versus residents born in Spain.

	Born in Spain			Immigrant		
Migratory Status	ORc	(95% CI)	1/Orc	ORa	(95% CI)	1/Ora
Born in Spain	1			1		
Immigrant	0.22	(0.09–0.53) *	4.52	0.07	(0.29–0.02) *	13.64

ORa: odds ratio adjusted by sex, age, educational level, occupational social class, work hours per week, informal employment, shiftwork, physical demand, unforeseen salary expenses, and city. * *p* < 0.05.

**Table 4:** Association (crude odds ratio (ORc) and adjusted odds ratio (ORa) with 95% confidence intervals (95% CI)) of common mental disorders in immigrants by time of residence versus residents born in Spain.

Migratory Status and Time of Residence	ORc	(95% CI)	1/ORc	ORa	(95% CI)	1/ORa
Born in Spain	1			1		
Immigrant: >15 years of residence	0.55	(2.25–0.14)	1.81	0.23	(1.50–0.03)	4.41
Immigrant: 11–15 years of residence	0.19	(0.66–0.06) *	5.18	0.06	(0.26–0.01) *	17.25
Immigrant: 1–10 years of residence	0.11	(0.63–0.02) *	8.96	0.06	(0.36–0.01) *	17.50

ORa: odds ratio adjusted by sex, age, educational level, occupational social class, works hours per week, informal employment, workday, physical demand, unforeseen salary expenses, and city. * *p* < 0.05.
